# Impaired White Matter Connections of the Limbic System Networks Associated with Impaired Emotional Memory in Alzheimer's Disease

**DOI:** 10.3389/fnagi.2016.00250

**Published:** 2016-10-27

**Authors:** Xiaoshu Li, Haibao Wang, Yanghua Tian, Shanshan Zhou, Xiaohu Li, Kai Wang, Yongqiang Yu

**Affiliations:** ^1^Department of Radiology, The First Affiliated Hospital of Anhui Medical UniversityHefei, China; ^2^Department of Neurology, The First Affiliated Hospital of Anhui Medical UniversityHefei, China

**Keywords:** Alzheimer disease, diffusion tensor imaging, emotional enhancement of memory, mild cognitive impairment, tract-based spatial statistics, voxel-based morphometry

## Abstract

**Background:** Discrepancies persist regarding retainment of emotional enhancement of memory (EEM) in mild cognitive impairment (MCI) and early Alzheimer's disease (AD) patients.In addition, the neural mechanisms are still poorly understood, little is known about emotional memory related changes in white matter (WM).

**Objective:** To observe whether EEM is absent in amnestic MCI (aMCI) and AD patients, and to investigate if emotional memory is associated with WM connections and gray matters (GM) of the limbic system networks.

**Methods:** Twenty-one AD patients, 20 aMCI patients and 25 normal controls participated in emotional picture recognition tests and MRI scanning. Tract-based spatial statistics (TBSS) and voxel-based morphometry (VBM) methods were used to determine white and gray matter changes of patients. Fourteen regions of interest (ROI) of WM and 20 ROIs of GM were then selected for the correlation analyses with behavioral scores.

**Results:** The EEM effect was lost in AD patients. Both white and gray matter of the limbic system networks were impaired in AD patients. Significant correlations or tendencies between the bilateral uncinate fasciculus, corpus callosum (genu and body), left cingulum bundle, left parahippocampal WM and the recognition sensitivity of emotional valence pictures, and significant correlations or tendencies between the splenium of corpus callosum, left cingulum bundle, left crus of fornix and stria terminalis and the recognition sensitivity of EEM were found. The volume of left amygdala, bilateral insula, medial frontal lobe, anterior and middle cingulum gyrus were positively correlated with the recognition sensitivity of emotional photos, and the right precuneus was positively correlated with the negative EEM effect. However, the affected brain areas of aMCI patients were more localized, and aMCI patients benefited only from positive stimuli.

**Conclusion:** There are impairments of the limbic system networks of AD patients. Damaged WM connections and GM volumes of those networks are associated with impaired emotional memory and EEM effect in AD patients.

## Introduction

Alzheimer's disease (AD) is the most common type of dementia in senile individuals, characterized by episodic memory impairment in the incipient stage, and a subsequent progressive and irreversible cognitive decline at later stages. Mild cognitive impairment (MCI) is a subjective cognitive impairment which initially, does not affect social functioning or activities associated with daily living. Impairment is usually first recognized by patients or their relatives, and confirmed with neuropsychological testing (Ewers et al., 2011). MCI is an umbrella term for a heterogeneous group of cognitive impairments; however amnestic MCI (aMCI) in particular, is thought to be a transition stage between normal aging and AD (Jicha et al., 2006; Whitwell et al., 2008; Mitchell and Shiri-Feshki, 2009).

Literature regarding structural and metabolic imaging in AD has highlighted several abnormalities in cortical and subcortical gray matter (GM), including areas in the hippocampus, parahippocampus gyrus, amygdala, entorhinal cortex, posterior cingulate cortex, and thalamus (Nestor et al., 2003; Whitwell et al., 2008; Ewers et al., 2011; Liu et al., 2014; Delli Pizzi et al., 2016; Pini et al., 2016). Many of these areas or subdivisions of these areas, such as the medial temporal lobe (MTL), anterior thalamus, mammillary bodies and posterior cingulate are thought to be strongly anatomically inter-connected via a limbic-diencephalic network termed the Papez circuit (Acosta-Cabronero and Nestor, 2014). The Papez circuit constitutes a group of neural projections involved in memory and emotional expression that originates in the cortex, and subsquently communicates with the hippocampal formation and mammillary bodies which in turn send projections to the anterior thalamic nuclei where information is relayed the cortex of cingulate gyrus (Vann and Nelson, 2015). Besides the circuit of Papez, Yakovlev proposed another limbic network which was consisted of the orbitofrontal cortex, insula, amygdala, and anterior temporal lobe, as an underlying pathway for emotion and motivation (Yakovlev, 1948). Maclean concluded that the limbic cortex, together with the limbic subcortical structures, is a functionally integrated system interconnected by short- and long-range fiber bundles (Maclean, 1949, 1952). Recently, a revised model was proposed by Catani to give a better and more comprehensive understanding of the limbic system. In Catani's model, there were three distinct but partially overlapping networks of the limbic system: the hippocampal-diencephalic and parahippocampal-retrosplenial network, the temporo-amygdala-orbitofrontal network, and the medial default-mode network, each responsed for different cognitive behaviors (Catani et al., 2013). In a recent study, higher mean diffusivity (MD) values were found bilaterally in the thalamic sub-regions projecting to temporal cortex, showing that the anterior nuclei projecting to temporal lobe were damaged in AD patients (Delli Pizzi et al., 2014). In fact, it has been proposed that AD was associated with the degeneration of the limbic cortices and their white matter (WM) connections (Acosta-Cabronero et al., 2010).

Because episodic memory loss is the hallmark of AD, it is necessary to understand different dimensions of that memory loss, and whether any episodic memory components, such as emotional memory, are relatively preserved in the disease progression. Normally, emotional stimuli are more easily remembered than neutral stimuli, a phenomena known as emotional enhancement of memory (EEM) (Hamann, 2001). Emotional stimuli attract more attention and are processed more fully than neutral stimuli, which in turn, enhances memory by increasing the distinctiveness with which they are encoded (Ochsner, 2000). The EEM effect exists in normal aging (Denburg et al., 2003; Murphy and Isaacowitz, 2008). On the contrary, studies have indicated that individuals with AD have impaired EEM following emotional stimuli. Hamann et al. (2000) showed that AD patients had impaired emotional memory in recall tests after viewing negative pictures only, but impairment of EEM was evident for both positive and negative pictures in the recognition task. Using recognition tests, other researchers have also found that AD patients do not retain EEM following emotional pictorial stimuli (Abrisqueta-Gomez et al., 2002; Landre et al., 2013). Specifically, Chainay et al. (2014) found impaired EEM of positive and negative pictures in AD patients after both intentional and incidental encoding. Additionally, AD patients also show disrupted EEM in recall tasks for emotional words, neutral words embedded in emotional context, and negative verbal information (Kensinger et al., 2002, 2004). Nevertheless, there are discrepancies in regard to whether the EEM effect is retained in patients with MCI and early AD. Although, some researchers have found that aMCI patients exhibit normal EEM in immediate recall tests (Nieuwenhuis-Mark et al., 2009), others have found that patients with aMCI do not retain EEM following visualization of emotional pictures (Parra et al., 2013; Wang P. et al., 2013). Sava et al. (2015) demonstrated that AD patients had a positive memory bias when the stimulus was sufficiently rich and deep encoded to the individual, and when support was provided the time of retrieval such as cued recall or recognition tasks. Another free-recall study highlighted the impaired EEM for verbal stimuli but preserved EEM for non-verbal declarative memory in patients with mild AD (Baran et al., 2014). These discrepancies may be a result of different materials used to elicit the emotional response, different arousal of stimuli, and also different encoding and retrieval methods used in each specific study. Alternatively the inconsistency may be due to the composition of patients with MCI and mild AD, which constitute a heterogeneous population with various possible levels of EEM preservation, depending on the state of their EEM-related brain structures.

Since the limbic system has been considered to play important roles in memory, emotion, spatial orientation, visceral sensation and behavior and associated with pathophysiology of many diseases (Catani et al., 2013; Guo et al., 2014; Liu et al., 2015). There might be some associations between the limbic system and emotional memory. Previous studies have focused on task related functional magnetic resonance imaging (MRI) or GM changes relating to EEM of AD patients (Dolcos et al., 2005; Perrin et al., 2012; Kumfor et al., 2013; Landre et al., 2013; Parra et al., 2013; Philippi et al., 2015), however, little is known about emotional memory related changes in WM. Thus, the purpose of the present study was to investigate whether the EEM is absent in patients with aMCI and AD, and whether emotional memory is related to WM connections of the limbic system. Our primary hypothesis was that the WM tracks of the limbic system would be impaired in AD patients, and this impairment would have a detrimental effect on their emotional memory and EEM. In regards to patients with aMCI, we hypothesized that any damage to the WM tracks would be more localized and less severe and therefore limit the extent of loss of EEM in this cohort.

## Materials and methods

### Participants

A total of 66 right-handed participants (21 AD patients, 20 aMCI patients and 25 normal controls (NCs)) took part in this experiment. The AD and aMCI patients were recruited from the Dysmnesia Outpatient Department at the 1st Affiliated Hospital of Anhui Medical University, Anhui Province, China. The NCs were recruited from the local community or were the spouses of the patients in the study. All experimental procedure were approved by the Medical Research Ethics Committee of the 1st Affiliated Hospital of Anhui Medical University. All subjects gave written informed consent in accordance with the Declaration of Helsinki.

The diagnosis of AD fulfilled the Diagnostic and Statistical Manual of Mental Disorders 4th Edition criteria for dementia and the revised National Institute of Neurological and Communicative Disorders and Stroke/Alzheimer Disease and Related Disorders Association (NINCDS-ADRDA) criteria for possible or probable AD: meets criteria for dementia, and has the following characteristics—insidious onset, clear-cut history of worsening of cognition by report or observation, the initial and most prominent cognitive deficits are evident on history and examination in amnestic presentation or non-amnestic presentations such as language, visuospatial and executive dysfunction (Mckhann et al., 2011). Mini-Mental State Examination (MMSE) score was less than 24. The Clinical Dementia Rating (CDR) score ranged from 0.5 to 2. The exclusion criteria are: history of sudden onset, early occurrence of gait disturbances, seizures and behavioral changes; focal neurological features including hemiparesis, sensory loss, visual field deficits; early extra-pyramidal signs and other severe disorders such as trauma, major depression, severe cerebrovascular disease, toxic and metabolic abnormalities (Dubois et al., 2007).

All MCI subjects were characterized as aMCI. aMCI was diagnosed according to the recommendations from the NINCDS-ADRDA criteria: complaints of memory loss and/or other cognition changes by patient, family or physician, in comparison with the person's previous level; evidence of lower performance in one or more cognitive domains that is greater than would be expected for the patient's age and educational background; preservation of independence in functional abilities and not demented (Albert et al., 2011). MMSE score >24; the CDR score of 0.5. The exclusion criteria are the same with AD patients.

The NCs were identified as: cognitively normal; no neurological or psychiatric disorders; no psychoactive medication; MMSE score of 28 or higher; and the CDR score was 0. However, as the presence of mild to moderate WM hyper-intensities (WMH) is a common accompaniment of aging and neurodegenerative diseases (Wardlaw et al., 2013), this was not considered as an exclusive criterion for all the participants (Liu et al., 2011). However, we graded the WMH according to the Fazekas scale (Fazekas et al., 1987) on the basis of visual assessment both periventricular (0 = absent, 1 = caps or pencil lining, 2 = smooth halo, 3 = irregular periventricular hyper-intensities extending into deep white matter) and subcortical areas (0 = absent, 1 = punctuate foci, 2 = beginning confluence of foci, 3 = large confluent areas). The total Fazekas score was calculated by adding the periventricular and subcortical scores together (Helenius et al., 2016), and was regressed out in the following statistics models. All the detailed background information regarding patient criteria can be found in Table 1.

**Table 1:** **Demographics and cognitive features of the patients and normal controls**.

	**AD group**	**MCI group**	**NC group**	**Statistic values**	***P*-values**
Number	21	20	25		
Age(years, *x* ± *s*)	68.19 ± 9.07	68.15 ± 8.67	64.52 ± 6.44	1.604^a^	0.209
Gender(male/female)	8/13	9/11	10/15	0.216^b^	0.898
Education time(years, *x* ± *s*)	9.24 ± 4.43^*^	12.05 ± 4.48	12.88 ± 2.98	5.148^a^	0.008
WMH score	1.90 ± 0.94	1.55 ± 1.14	1.68 ± 0.90	0.674^a^	0.513
MMSE scores(*x* ± *s*)	16.57 ± 4.18	26.45 ± 1.73	28.92 ± 1.29	132.814^a^	<0.001
ADL	32.89 ± 12.62	21.39 ± 3.07		3.856	0.001
GDS(deterioration)	3.91 ± 0.64	2.90 ± 0.31		5.934	<0.001
AVLT(immediate)	3.14 ± 1.51	5.60 ± 1.72		4.069	<0.001
AVLT(delayed)	0(0)	5(5)		4.000^c^	<0.001
DS(forward)	5.24 ± 1.92	6.57 ± 1.22		2.250	0.032
DS(backward)	2.94 ± 1.20	3.79 ± 0.89		2.184	0.037
GDS(depression)	4.71 ± 3.65	3.53 ± 2.50		1.095	0.282

a One-way ANOVA F-values;

* post-hoc comparisons with significant statistic difference compared with the other 2 groups.

b Pearson Chi-square (2 tails) λ^2^-value.

c Rank-sum test U-value; others are 2-Independent t-test t-values. WMH, white matter hyper-intensities; MMSE, Mini-Mental State Examination; ADL, Activities of Daily Living scale; GDS(deterioration), Global Deterioration Scale; AVLT, Auditory Verbal Learning Test; DS, Digit Span Test; GDS(depression), Geriatric Depression Scale. p < 0.05 means significant statistic difference.

### Emotional memory behavior

This behavioral test was divided into two phases, an encoding phase and a retrieval phase. For the encoding phase, the stimuli consisted of 90 color photographs containing an equivalent number of images based on emotional valence (30 negative, 30 neutral, 30 positive). Each photo contained various images of people and all stimuli that appeared in the encoding phase were used during the retrieval phase. In addition, a further 90 photographs (30 negative, 30 neutral, 30 positive) were used during the retrieval phase. Thus, in all, participants always saw 90 stimuli in the encoding phase and 180 stimuli in the retrieval phase (90 targets, 90 distractors). The photographs were selected from the International Affective Picture System, with two independent properties, each ranging from 1 to 9, where 1 corresponds to very negative on valence scale and to low emotional arousal on the arousal scale, and 9 corresponds to very positive on valence scale and highly arousing on the arousal scale. Mean rating for negative valence was 4.0 or less; mean rating for positive valence was 6.0 or more; mean rating for neutral valence was between 4.5 and 5.5; and mean rating for arousal was between 2 and 8, respectively. The tasks were performed using the DMDX 3.01 software (http://www.u.arizona.edu/~kforster/dmdx/dmdx.htm) and a stimulus was displayed on a laptop screen screen for 2500 ms with an interval time of 500 ms between each display.

During the encoding phase, participants were asked immediately to determine whether the number of people in the photo was greater than two (categorization task). The categorization task was employed to make sure that the participants visualized images in the photo in a meaningful way. The retrieval phase was done immediately after the encoding phase and participants were not told of the subsequent retrieval task ahead of the encoding phase. During the retrieval phase, participants were asked to decide immediately whether the picture had been seen previously (recognition task).

### MRI

MRI scans were performed on a General Electric HDxt 3.0T MRI scanner with 8 channel head-coil (General Electric, Waukesha, WI, USA). The imaging protocol included diffusion tensor imaging (DTI), T1-weighted three-dimensional structure sequence, axial T2-weighted and fluid-attenuated inversion recovery images. Subjects with abnormalities other than atrophy or leukoaraiosis were excluded. DTI data were collected using spin echo single shot echo planar imaging sequence (repetition time (TR)/echo time (TE)/number of excitations = 8000 ms/87 ms/3; Matrix 128 × 128, field of view (FOV) 240 × 240 mm, 30 contiguous axial slices with slice thickness of 4 mm) with diffusion-sensitizing gradient orientations along 30 non-collinear directions (*b* = 1000 s/mm^2^) and with one scan without diffusion weighting (*b* = 0 s/mm^2^, b_0_). The acquisition time was 12 min 32 s.

T1-weighted three-dimensional structure data were collected using fast spoiled gradient recalled echo sequence (TR/TE/inversion time (TI) = 7 ms/2.9 ms/900 ms; Matrix 256 × 256, FOV 240 mm × 226 mm, slice thickness of 1.2 mm without intervals). The acquisition time was 6 min 7 s.

### DTI data preprocessing

The raw DTI data were transformed from dicom format to nifti format using Mricron software. The Functional Magnetic Resonance Imaging of the Brain (FMRIB) Software Library (FSL) (http://fsl.fmrib.ox.ac.uk/fsl/fslwiki/FSL) was employed to process and analyze the DTI data. Firstly, each diffusion-weighted volume was affine-aligned to its corresponding b_0_ image using the FMRIB's linear image registration tool, corrected for possible motion artifacts and eddy-current distortions. Secondly, extra-cranial tissues were removed using the brain-extraction tool with a fractional threshold of 0.2, and robust brain center estimation algorithm. FMRIB's diffusion toolbox was then used to fit the tensor and compute the fractional anisotropy (FA), MD, axial diffusivity (λ1) and radial diffusivity (RD) at each brain voxel. Then the tract-based spatial statistics (TBSS) steps were conducted. The FA image of each subject was aligned to a pre-identified target FA image (FMRIB58 FA_1 mm) by non-linear registrations, then transformed into the Montreal Neurological Institute 152 template (1 × 1 × 1 mm) by affine registrations. The mean FA image and mean FA skeleton were created from all subjects. A mean FA skeleton mask was generated using an FA threshold of 0.2 and individual subjects FA images were projected onto the skeleton. Data for MD, λ1 and RD were also generated by applying the above FA transformations to the additional non-FA diffusivity maps and projecting them onto the skeleton.

### T1-weighted three-dimensional structure data preprocessing

GM atrophy was assessed using modulated voxel-based morphometry (VBM) in Statistical Parametric Mapping 8 (SPM8; http://www.fil.ion.ucl.ac.uk/spm/software/spm8). The three-dimensional structure data were segmented into GM, WM and cerebrospinal fluid using the VBM8 software. Those components were normalized to the standard Montreal Neurological Institute space by affine and non-linear registrations and a dartel algorithm. The GM density was then transformed into the relative GM volume (GMV) after non-linear modulation. SPM8 software was used for smooth imaging with an 8 mm Gaussian kernel.

### Statistical analysis

#### Emotional memory behavior test

For every participant, we calculated the indices of correct recognition rate of previously seen stimuli in the encoding phase (called Hits for short) and false alarms rate (FAs). We then computed indices of sensitivity (d′) and response criterion (C), corresponding to sensitivity and bias measures derived from the signal detection theory. As participants differed significantly in the level of education, analysis of 3 × 3 mixed-factorial co-variances were performed on each of these four indices, with group (AD, MCI, and NC) as a between-subject factor, valence (negative, neutral, and positive) as a within-subject factor, and the education level of participants as a covariate. Although the age, gender and WMH scores were not significantly different among the three groups, they were also added as covariates for the consistency with imaging data analyses. The least significant difference (LSD) correction was used for *post-hoc* multiple comparisons. We excluded four AD and five MCI patient's behavior results because of limited understanding of the test or overall non-engagement in the task processes.

#### WM damage

TBSS statistical analyses were performed to compare FA, MD, λ1, and RD, respectively, using a permutation-based nonparametric statistical method (“randomized,” part of the FSL). Firstly, an *F*-test was defined in order to determine the different WM areas among the three groups, after which *post-hoc* analyses were done to search the specific differences between the AD and NC groups, the aMCI and NC groups, and the AD and aMCI groups, respectively. The number of permutations was set at 5000. Analyses were adjusted for age, gender, education level and WMH scores. The resulting statistical maps had a threshold of *p* < 0.05, and family-wise error (FWE) was corrected for multiple comparisons using the threshold-free cluster enhancement (TFCE) option, which controls the rate of type I errors.

#### GM damage

In order to be comparable with the WM results, VBM statistical analyses were also conducted using the permutation-based nonparametric statistical method. As mentioned above, an *F*-test was initially defined to determine the different GM areas among the three groups, and then *post-hoc* analyses were done to search the specific differences between any two groups: AD vs. NC, aMCI vs. NC, and AD vs. aMCI. Significance level was also set at *p* < 0.05 and FWE corrected for multiple comparisons using the TFCE option. The analyses were also adjusted for age, gender, education level and WMH scores. The number of permutations was set at 5000.

#### Correlation analysis

The International Consortium of Brain Mapping (ICBM) DTI-81 WM labels atlas was used to guide the identification of WM tracts of interest: the genu, body and splenium of corpus callosum; fornix (column and body of fornix); bilateral anterior limb of internal capsule, bilateral cingulum (cingulate gyrus), bilateral cingulum (parahippocampus), bilateral fornix (crus)/stria terminalis and bilateral uncinate fasciculus, with a total of 14 regions of interest (ROI) selected. Meantime, the Anatomical Automatic Labeling (AAL) atlas was used to guide the identification of GM brain areas of interest: bilateral hippocampus, parahippocampal gyrus, amygdala, insula, posterior cingulate gyrus, middle cingulate gyrus, anterior cingulate gyrus, medial frontal lobe, precuneus, and thalamus. Correlation analyses between each parameter (FA, MD, λ1, RD, and GMV) and the index d′ of emotional valence pictures [i.e., d′(negative), d′(positive)) and EEMd′ (EEMd′(negative) = d′(negative) − d′(neutral), EEMd′(positive) = d′(positive) − d′(neutral)] were performed using the SPSS 10.0 software.

## Results

### Emotional memory behavior

For the indices Hits and FAs, the main effects of group were significant [*F*_(2, 50)_ = 9.102, *p* < 0.001; and *F*_(2, 50)_ = 14.69, *p* < 0.001, respectively]. NCs showed the highest hit rate (mean_Hits_ = 0.63) and the lowest false alarms rate (mean_FAs_ = 0.18), which is just on the contrary of AD patients (mean_Hits_ = 0.44, mean_FAs_ = 0.45). On one hand, aMCI patients showed similar false alarms rate (mean_FAs_ = 0.21, *p* = 0.56) with NCs, on the other hand, they had similar hit rate (mean_Hits_ = 0.47, *p* = 0.532) with AD patients. The main effect of emotion and the valence × group interaction were not significant (for all comparisons, *p* > 0.05).

**Figure 1:**
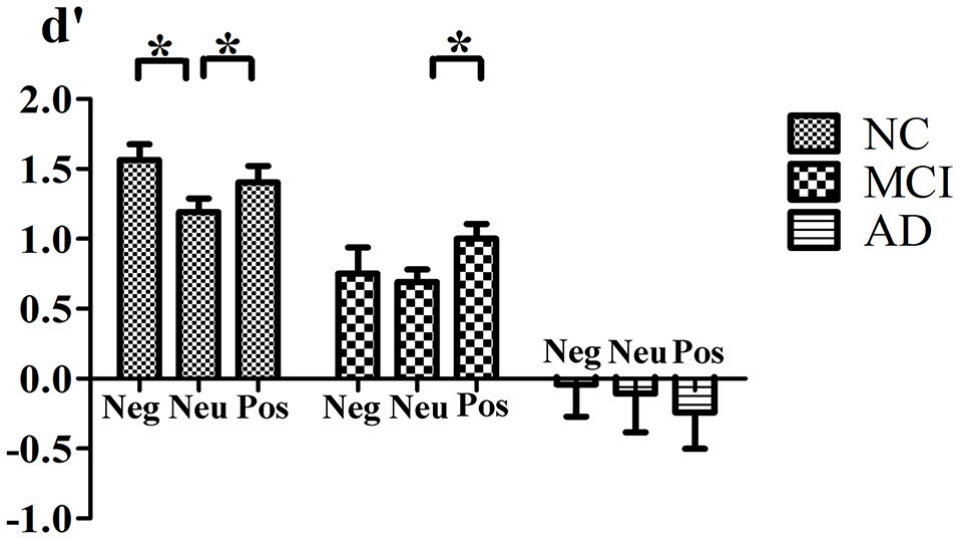
**Interaction of Valence × Group**. Mean value of d′ index for negative, neutral and positive stimuli for normal control, MCI and AD patients. ^*^Indicates significant statistic difference. Bars represent standard error. Abbreviations: Neg, negative; Neu, neutral; Pos, postitive.

For the index d′, the main effect of group [*F*_(2, 50)_ = 21.40, *p* < 0.001] was significant. NCs (mean = 1.36) discriminated previously seen stimuli significantly better than aMCI and AD patients (mean = 0.87, *p* = 0.028, and mean = −0.15, *p* < 0.001, respectively) and aMCI patients discriminated these stimuli better than AD patients (*p* < 0.001). The main effect of emotion [*F*_(2, 49)_ = 1.349, *p* = 0.269, Pillai's Trace] was not significant. However, the valence × group [*F*_(4, 100)_ = 2.620, *p* = 0.039, Pillai's Trace] interaction was significant. NCs discriminated both previously seen positive (*p* = 0.023) and negative (*p* = 0.002) stimuli better than that of neutral ones. However, the difference between positive and negative stimuli was not significant (*p* = 0.144). For aMCI patients, previously seen positive stimuli were better discriminated than that of neutral ones (*p* = 0.031), however the difference between negative and neutral stimuli (*p* = 0.789) or between positive and negative stimuli (*p* = 0.088) was not significant. No difference was found in AD patients in discrimination of old stimuli according to emotional valence (for all pairwised comparisons, *p* > 0.05; Figure 1).

For the index C, both the main effect of group [*F*_(2, 50)_ = 0.77, *p* = 0.47] and the main effect of emotion [*F*_(2, 49)_ = 0.39, *p* = 0.679, Pillai's Trace] were not significant. In addition there was no significant valence × group interaction [*F*_(4, 100)_ = 0.427, *p* = 0.789, Pillai's Trace)].

### WM damage

WM differences in various brain regions was widespread among the three groups, including the corpus callosum, cingulum, fornix, corona radiata, thalamic radiation, internal capsule, external capsule, superior longitudinal fasciculus, inferior longitudinal fasciculus, inferior fronto-occipital fasciculus, superior fronto-occipital fasciculus and uncinate fasciculus (*p* < 0.05, FWE corrected).

#### AD vs. NC

**Figure 2:**
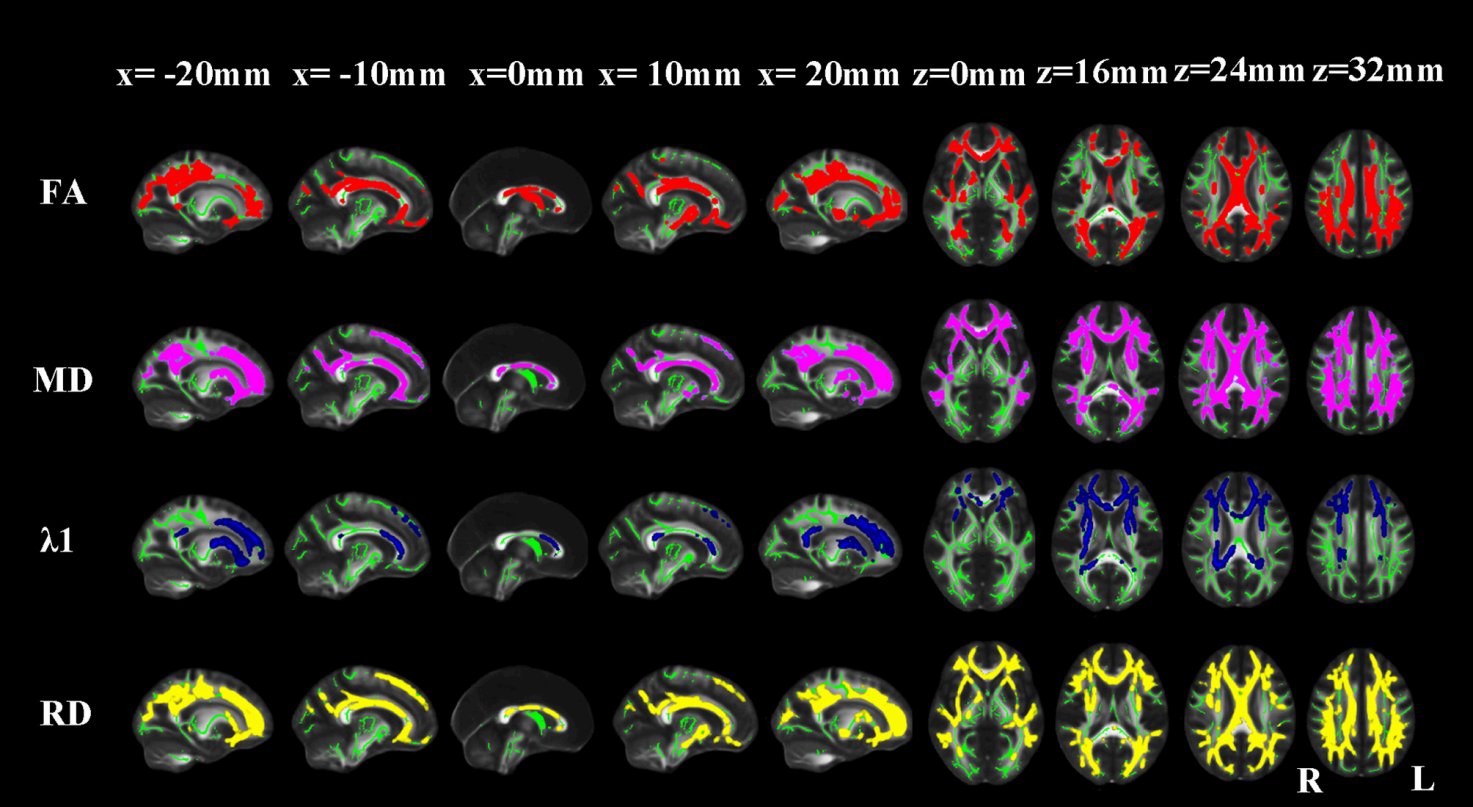
**TBSS results of the diffusion indices between the AD and NC groups**. Green represents the mean white matter skeleton of all subjects; red, purple, blue, and yellow represented the regions with reduced FA, increased MD, increased λ1 and increased RD respectively in the AD patients (*p* < 0.05, FWE corrected).

Compared with NCs, AD patients showed an approximate symmetrical pattern of significantly reduced FA as well as increased MD and RD in the genu, body and splenium of corpus callosum; retrolenticular part of internal capsule; anterior, superior and posterior corona radiata; posterior thalamic radiation; sagittal stratum (include inferior longitudinal fasciculus and inferior fronto-occipital fasciculus); external capsule; cingulum (cingulate gyrus); the crus of fornix and stria terminalis; superior longitudinal fasciculus; uncinate fasciculus, right anterior and posterior limb of internal capsule and right anterior thalamic projection. Extra FA decreases were found in the column and body of the fornix. Extra MD or λ1 or RD increases were found in the left anterior and posterior limb of internal capsule; left anterior thalamic projection and bilateral superior fronto-occipital fasciculus. The pattern of increased λ1 in WM areas appeared more localized than the other three indices (Figure 2).

#### aMCI vs. NC

**Figure 3:**
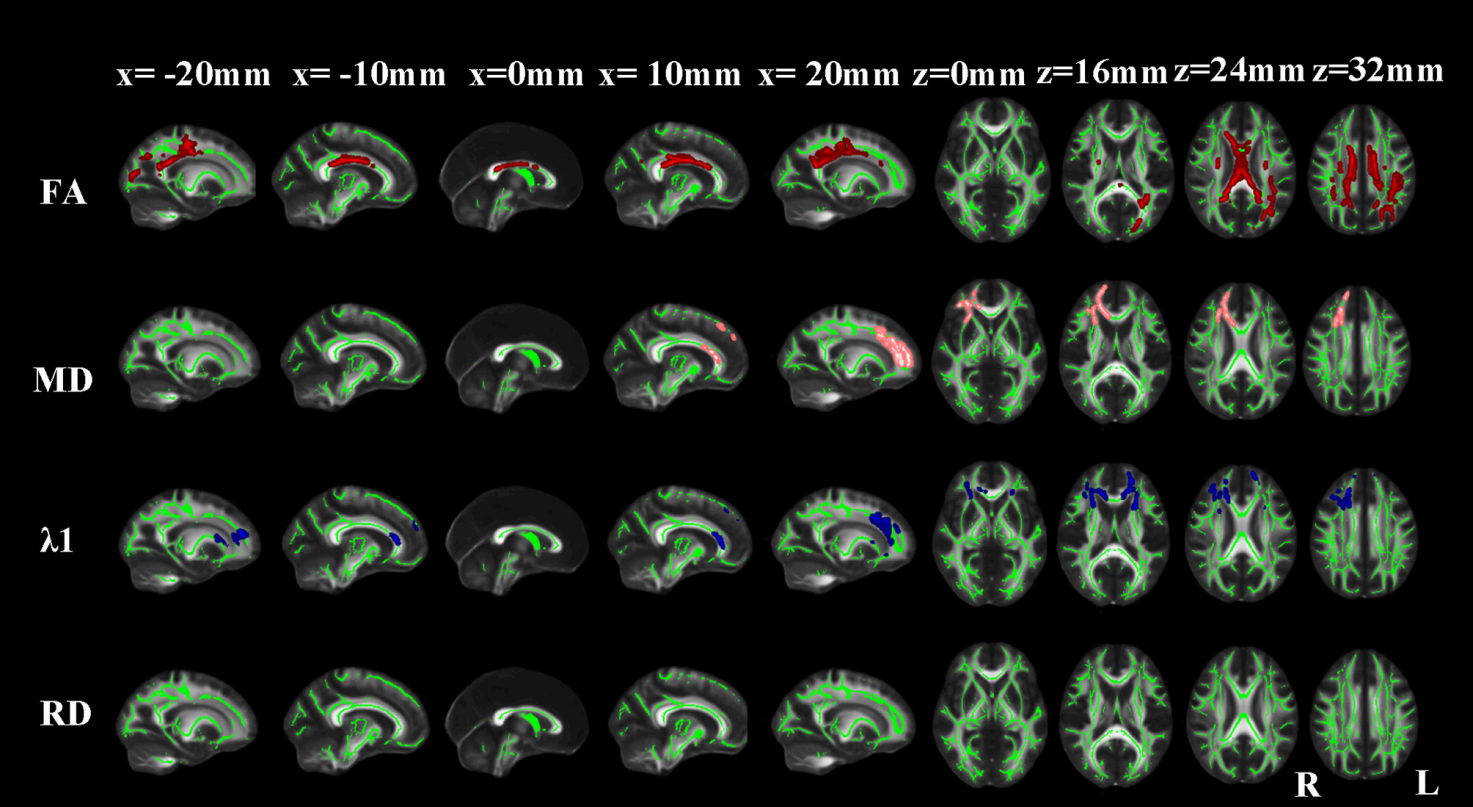
**TBSS results of the diffusion indices between the MCI and NC groups**. Green represents the mean white matter skeleton of all subjects; red, pink, and blue represented the regions with reduced FA, increased MD and increased λ1 respectively in the MCI patients. No changes of RD were found (*p* < 0.05, FWE corrected).

Compared with NCs, aMCI patients showed significantly reduced FA in the corpus callosum, bilateral superior and posterior corona radiata, bilateral cingulum (cingulate gyrus), bilateral superior longitudinal fasciculus, right posterior limb of internal capsule, right external capsule, right anterior corona radiata, right uncinate fasciculus and left posterior thalamic radiation. Extra λ1 increases were found in the bilateral anterior limb of internal capsule, bilateral anterior thalamic projection, right superior fronto-occipital fasciculus, left external capsule and left anterior corona radiata (Figure 3).

#### AD vs. aMCI

Compared with aMCI, AD patients showed significantly reduced FA as well as increased MD and RD in the left crus of fornix and stria terminalis, corpus callosum, bilateral anterior, superior and posterior corona radiata, posterior thalamic radiation, cingulum (cingulate gyrus), superior longitudinal fasciculus and left uncinate fasciculus, external capsule, sagittal stratum (include inferior longitudinal fasciculus and inferior fronto-occipital fasciculus), left retrolenticular part and posterior limb of internal capsule. Extra MD or λ1 or RD increases were found in the bilateral anterior limb of internal capsule and anterior thalamic projection, right posterior limb and retrolenticular part of internal capsule, right external capsule, right sagittal stratum (include inferior longitudinal fasciculus and inferior fronto-occipital fasciculus), right superior fronto-occipital fasciculus and right uncinate fasciculus. In particular, an increased RD in the right crus of fornix and stria terminalis was found in AD patients compared with aMCI patients.

### GM damage

**Figure 4:**
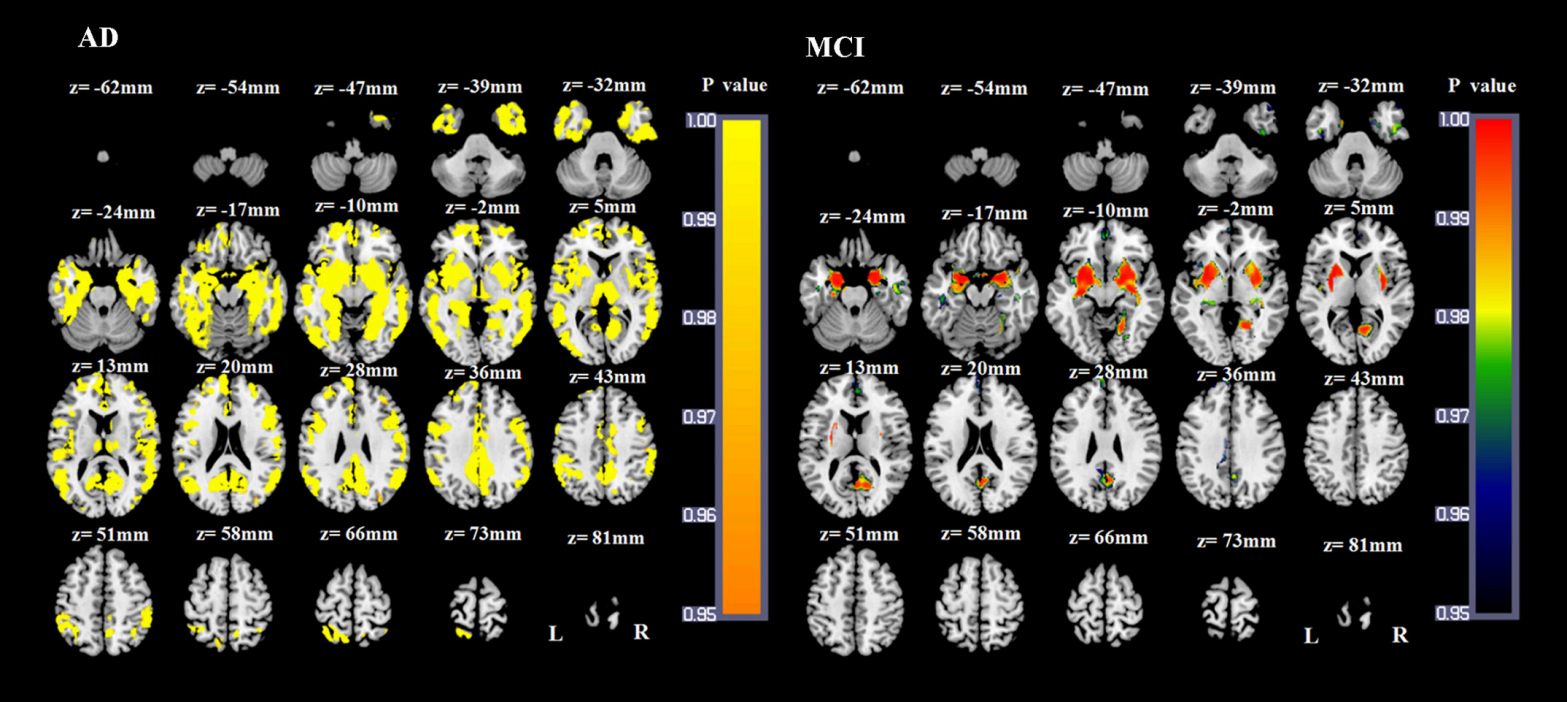
**VBM results of the atrophied brain areas of AD and MCI patients compared with NC groups (*p* < 0.05, FWE corrected)**.

Compared with NCs, AD patients showed a widespread pattern of GM atrophy, involving the bilateral hippocampus, parahippocampal gyrus, amygdala, insula, temporal gyrus, cuneus, precuneus, posterior cingulate gyrus, middle cingulate gyrus, anterior cingulate gyrus, obital and superior medial frontal lobe, frontal gyrus, fusiform gyrus, lingual gyrus, calcarine, angular gyrus, supramarginal gyrus, inferior occipital gyrus, middle occipital gyrus, bilateral putamen, pallidum, thalamus and right caudate nucleus (*p* < 0.05, FWE corrected; Figure 4). Compared with NCs, the atrophied GM areas of aMCI patients were concentrated in the bilateral amygdala, hippocampus, parahippocampal gyrus, insula, medial frontal lobe, precuneus, putamen, pallidum, left posterior cingulate gyrus, right fusiform, right lingual gyrus, and right calcarine (*p* < 0.05, FWE corrected; Figure 4). As the dementia progressed, AD patients showed more severe atrophied GM areas in the bilateral hippocampus, parahippocampal gyrus, amygdala, insula, temporal gyrus, cuneus, precuneus, fusiform gyrus, posterior cingulate gyrus, middle cingulate gyrus, left anterior cingulate gyrus, left superior medial frontal lobe, bilateral frontal gyrus, middle occipital gyrus and inferior occipital gyrus, calcarine, lingual gyrus, angular gyrus, putamen, thalamus and right caudate nucleus compared with aMCI patients (*p* < 0.05, FWE corrected).

### Correlation analysis

**Figure 5:**
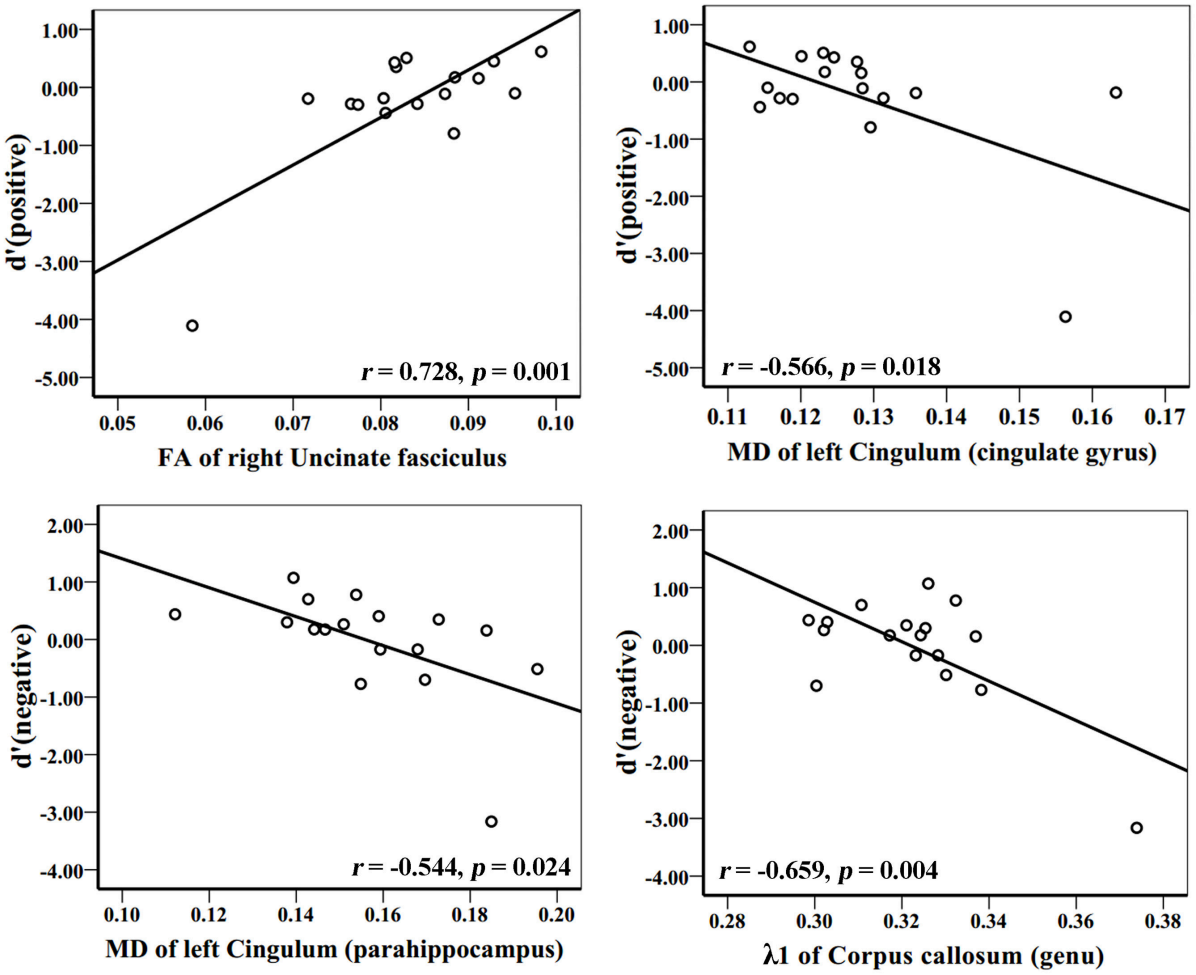
**Correlation analyses between d′ (positive), d′ (negative), and DTI indices in AD group patients**. The unit of MD or λ1 is 10^−3^mm^2^/s.

**Figure 6:**
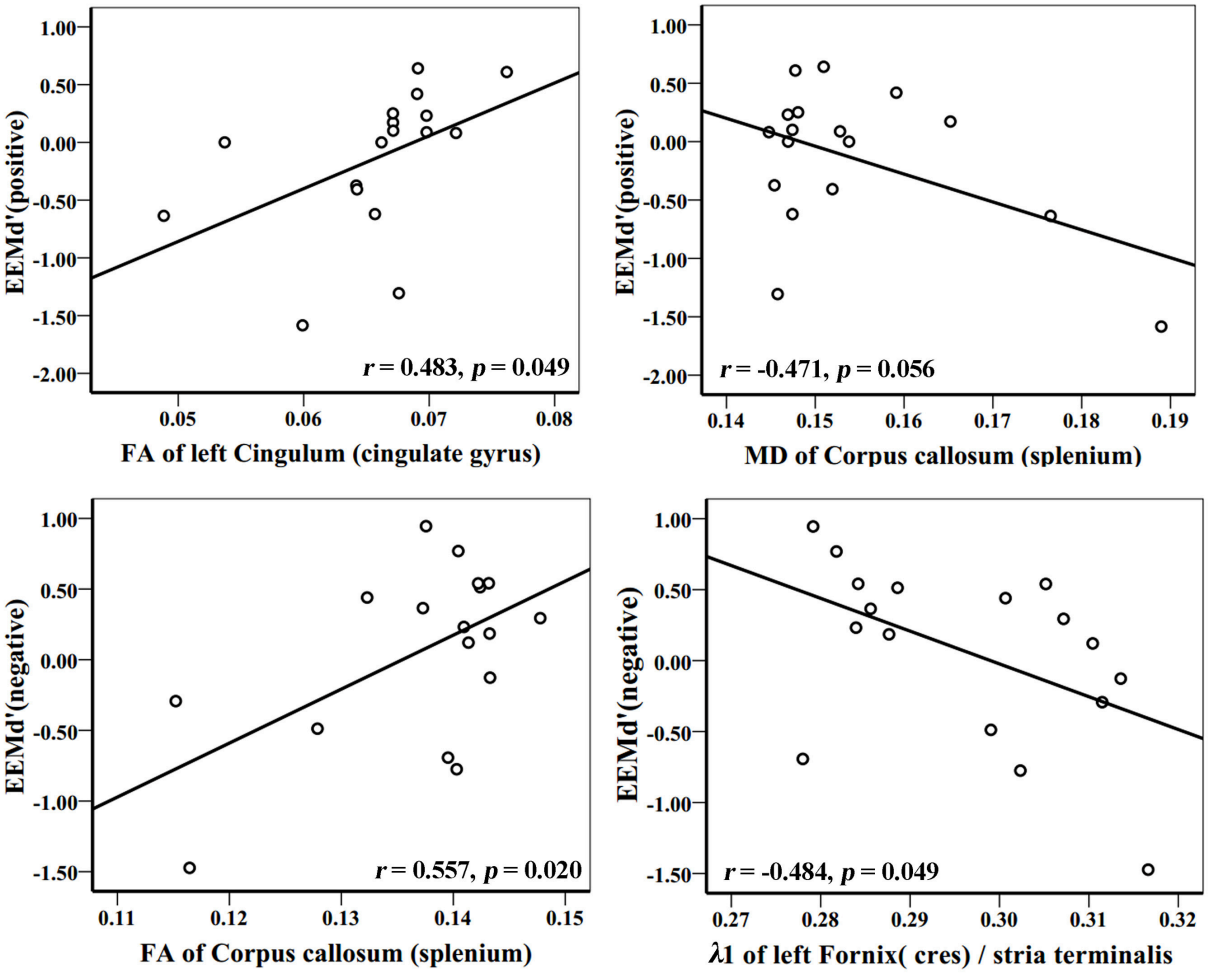
**Correlation analyses between EEMd′ (positive), EEMd′ (negative) and DTI indices in AD group patients**. The unit of MD or λ1 is 10^−3^mm^2^/s.

In the AD group, WM tracks of the hippocampal-diencephalic and parahippocampal-retrosplenial network (i.e., left cingulum bundle and parahippocampal WM); bilateral uncinate fasciculus and corpus callosum (genu and body) had statistically significant correlations or tendencies with the recognition sensitivity of emotional photos (Figure 5; Table 2). The splenium of corpus callosum, left cingulum bundle, left crus of fornix and stria terminalis had statistically significant correlations or tendencies with the EEMd′ (Figure 6; Table 2). The volume of left amygdala, bilateral insula, medial frontal lobe, anterior and middle cingulum gyrus were positively correlated with the recognition sensitivity of both negative and positive emotional photos, and the right precuneus was positively correlated with the EEMd′(negative). However, in aMCI patients, no significant correlations except the positive correlations between FA value of right uncinate fasciculus and d′(positive) (*r* = 0.555, *p* = 0.032), and between the volume of right amygdala and d′(positive) (*r* = 0.515, *p* = 0.049) were found.

**Table 2:** **Correlation analysis with emotional memory and EEM of AD patients**.

**Brain regions**	**d′(pos)**		**d′(neg)**		**EEMd′(pos)**		**EEMd′(neg)**	
	***r***	***p***	***r***	***p***	***r***	***p***	***r***	***p***
**FA**								
L_ uncinate fasciculus	0.721	0.001	0.681	0.003				
R_ uncinate fasciculus	0.728	0.001	0.726	0.001				
L_Cingulum (cingulate gyrus)	0.554	0.021	0.490	0.046	0.483	0.049		
L_Cingulum (parahippocampus)	0.472	0.056	0.528	0.029				
Corpus callosum (splenium)							0.557	0.020
**MD**								
R_ uncinate fasciculus	−0.571	0.017	−0.643	0.005				
L_Cingulum (cingulate gyrus)	−0.566	0.018	−0.521	0.032				
L_Cingulum (parahippocampus)			−0.544	0.024				
Corpus callosum (genu)			−0.529	0.029				
Corpus callosum (body)	−0.489	0.047						
Corpus callosum (splenium)					−0.471	0.056	−0.564	0.018
λ**1**								
L_ uncinate fasciculus	−0.572	0.016	−0.543	0.024				
Corpus callosum (genu)	−0.727	0.001	−0.659	0.004				
Callosum (body)	−0.533	0.027	−0.481	0.051				
L_Fornix(cres)/stria terminalis							−0.484	0.049
**RD**								
L_ uncinate fasciculus	−0.899	<0.001	−0.840	<0.001				
R_ uncinate fasciculus	−0.748	0.001	−0.786	<0.001				
L_Cingulum (cingulate gyrus)	−0.614	0.009	−0.551	0.022				
L_Cingulum (parahippocampus)	−0.482	0.049	−0.556	0.021				
Corpus callosum (genu)			−0.587	0.013				
Corpus callosum (splenium)							−0.585	0.014
**GRAY MATTER VOLUME**								
L_Amygdala	0.517	0.034	0.548	0.023				
L_Insula	0.575	0.016	0.584	0.014				
R_Insula	0.562	0.019	0.500	0.041				
L_Medial frontal lobe	0.646	0.005	0.691	0.002				
R_Medial frontal lobe	0.737	0.001	0.779	<0.001				
L_anterior Cingulum gyrus	0.662	0.004	0.689	0.002				
R_anterior Cingulum gyrus	0.690	0.002	0.726	0.001				
L_middle Cingulum gyrus	0.681	0.003	0.686	0.002				
R_middle Cingulum gyrus	0.594	0.012	0.613	0.009				
R_Precuneus							0.514	0.035

d′(pos), d′ (positive); d′(neg), d′ (negative); EEMd′(pos), EEMd′ (positive); EEMd′(neg), EEMd′ (negative).

## Discussion

Based on our findings, using pictures as stimuli, incidental encoding and recognition tasks, NC participants recognized previously seen stimuli better than aMCI and AD patients. Compared with NC group, aMCI patients showed decreased Hits but normal FAs, which indicated that the impaired episodic memory in amnesia appeared to affect the correct recognition rate firstly. Furthermore, for the index d′, only the NC group benefited from the emotional stimuli of both negative and positive images. aMCI patients benefited only from positive stimuli. AD patients didn't differ in terms of discrimination of old stimuli according to emotional valence. The results were agreed with our hypothesis in regards to emotional memory in AD and aMCI patients. The EEM effect is impaired in AD patients but preserved in aMCI patients to some extent. However, aMCI didn't discriminate old negative pictures better than that of neutral ones. One probable explanation is that aMCI patients may spontaneously focus their attention on positive information and process it in a more self-referential way because of their limited cognitive resources. Another explanation is that the atrophied amygdala volumes in aMCI patients may preferentially affect negative emotion processing. However, in this study, for the index Hits, there were no significant valence × group difference and no EEM effect, even in the NC group. Since the definition of Hits is the correct recognition rate of previously seen stimuli. It is well accepted that recognition relies on both familiarity and recollection (Yonelinas, 2001). In fact, there would be three kinds of psychological state, “remember,” “know,” or “guess,” when a picture was judged by someone as old. So, only Hits alone could not reflect the real discrimination, and this might influence the detection of EEM effect. What's more, here in this study, we conducted an incidental encoding procedure, in which less cognition resource was involved, leading a participant more likely to depend his/her answer on a sense of familiarity. Several behavioral studies have shown that the memory-enhancing effect of emotion specifically modulates recollection rather than familiarity (Dolcos et al., 2005; Wang P. et al., 2013). Thus, the EEM effect of hit rate might be contaminated due to greater proportion of familiarity-based response for all stimuli. Our results were partly consistent with Chainay et al. who did not observe EEM for Hits after incidental encoding either (Chainay et al., 2014). This may also suggest that the index d′ is more suitable and sensitive than the index Hits to reflect EEM effect.

According to the TBSS results, AD patients showed widespread WM impairments, including association fibers, commissural fibers and projection fibers, which indicated impaired WM connections both between and within hemispheres. In addition, the WM connections of the limbic system networks, including the column and body of fornix, bilateral crus of fornix and stria terminalis, cingulum bundles, anterior thalamic radiation, and uncinate fasciculus were impaired in AD patients. Consistent with the proposed functionally integrated viewpoint, VBM results from the present cohort confirmed that AD patients had GM atrophy of the limbic system, such as the MTL structures (i.e., amygdala, hippocampus, parahippocampal gyrus), thalamus, posterior cingulate gyrus, orbitofrontal and medial prefrontal cortex, and precuneus.

Firstly, the hippocampal-diencephalic and parahippocampal-retrosplenial network was impaired in AD patients. The hippocampal-diencephalic limbic circuit is connected through the fornix and mammillo-thalamic tract, and the parahippocampal-retrosplenial circuit is connected through the ventral cingulum (Catani et al., 2013). The hippocampus has long been considered as a central structure for episodic memory encoding, consolidation and retrieval. However, a role for the hipocampus in emotional control has become more widely appreciated recently. The fornix has played a fundamental role in linking MTL and diencephalon (Acosta-Cabronero and Nestor, 2014). Using tractography, it has been shown that the integrity of the fornix is linked to memory (Metzler-Baddeley et al., 2012). Nestor et al. found λ1 abnormalities in the fornix, parahippocampal WM, and anterior thalamus of an AD cohort (Nestor et al., 2003). Specific research targeting the fornix, cingulum bundle and corpus callosum, found significant cross-sectional and longitudinal tensor differences for all three structures (Keihaninejad et al., 2013). These major WM connections of the limbic system have been highlighted in the “early hypometabolic landscape” of AD. Secondly, the temporo-amygdala-orbitofrontal network was impaired in AD patients. The temporo-amygdala-orbitofrontal network is connected through the uncinate fasciculus, and is dedicated to the integration of visceral and emotional states with cognition and behavior (Catani et al., 2013). Previous studies have indicated that emotional memory is dependent on the integrity of the amygdala, and the interaction between the amygdala and other brain regions, such as the hippocampal formation, prefrontal cortex, sensory neocortex, striatum, and hypothalamic–pituitary–adrenal axis (Labar and Cabeza, 2006). The amygdala receives information related to the external environment from the sensory thalamus and sensory cortices, and is reciprocally connected with cortical regions, especially the hippocampus, and the midline and orbital prefrontal cortices (Janak and Tye, 2015). It has been proposed that EEM may be based on two different mechanisms related to the amygdala; one based on the influence of the amygdala on attention processes (attention-mediation hypothesis) and the other related to modulation of consolidation processes (consolidation-mediation hypothesis) (Hamann, 2001; Talmi et al., 2008; Hermans et al., 2014). In fact, the uncinate fasciculus links the amygdala and temporal lobe with the prefrontal lobe. It is critical for episodic memory. Some researches have indicated that the uncinate fasciculus was impaired in AD and MCI patients (Fujie et al., 2008; Yasmin et al., 2008; Kiuchi et al., 2009), and might be associated with impaired facial emotional recognition in aMCI (Fujie et al., 2008). Our results confirmed that the integrity of uncinate fasciculus was aslo associated with the discrimination of previous seen emotional pictures in AD patients. Thirdly, the medial default-mode network was impaired in AD patients. Actually, the medial regions of the default-mode network are inter-connected through the dorsal cingulum. It is well known that the cingulum is an important component of the limbic system, and can be divided into an anterior-dorsal component, which constitutes most of the white matter of the cingulate gyrus, and a posterior-ventral component running within the parahippocampal gyrus, retrosplenial cingulate gyrus, and posterior precuneus (Catani et al., 2013). The anterior cingulate/medial prefrontal cortex and the posterior cingulate/precuneus form the medial default-mode network. The DMN network is involved in introspection and active episodic memory (Barkhof et al., 2014). The majority of resting-state functional MR studies found reduced DMN connectivity in patients with AD but also in the earlier phase of MCI, even in unaffected carriers of familial AD and subjects with cognitive complaints (Greicius et al., 2004; Sheline and Raichle, 2013; Wang Y. et al., 2013). Consistent with previous DTI studies (Liu et al., 2011; Shu et al., 2011; Wai et al., 2014), our results indicated structures outside the limbic system, including the sagittal stratum (include inferior longitudinal fasciculus and inferior fronto-occipital fasciculus), superior longitudinal fasciculus, superior fronto-occipital fasciculus, corona radiata, posterior thalamic radiation and external capsule, were also affected in AD patients.

Moreover, by combining these microstructure impairments of the limbic system networks with emotional memory scores, we found that the uncinate fasciculus, corpus callosum, left cingulum bundles, left parahippocampal WM, left crus of fornix and stria terminalis had correlations with the index d′ of emotional valence pictures or EEMd′ in AD patients, thus supporting our previous hypothesis that WM connections of the limbic system were associated with emotional memory and EEM impairment. It tends to indicate that the few AD patients still benefiting from the effect of emotional valence on memory are the ones who have more intact WM fiber connections, even though most patients do not benefit from this effect at all. Additionally, the GMV of limbic system networks such as the amygdala, insula, medial frontal lobe, and cingulum gyrus, were also associated with emotional memory in AD patients, which exactly reveals the WM and GM functional integrity theory of the limbic system networks.

By contrast, the atrophied brain areas of aMCI were more limited, nevertheless, structures of the MTL, precuneus and medial prefrontal cortex were already affected. Although, several WM tracks associated with the limbic system were also affected in aMCI patients, these impairments were more localized and less severe, and no significant correlations except the positive correlations between the FA value of right uncinate fasciculus and the d′(positive), and between the volume of right amygdala and d′(positive) were found in aMCI patients. One probable explanation is that the WM impairments in aMCI patients were still too subtle to cause disfunctions of those fibers. Another possible explanation is the higher brain reserve of aMCI patients. Here in our study, aMCI patients had a higher educational level and more localized impaired brain areas than AD patients. It has been hypothesized that “brain reserve” may account for inter-individual differences in the recruitment of neural networks and cognitive processes, and may thus compensate for age-related brain dysfunction (Freret et al., 2015). In fact, one study has revealed that the preserved executive functioning in MCI patients was associated with a higher education time (Andrejeva et al., 2016). Another 12-month follow-up study has demonstrated that higher cognitive reserve contributed to protecting against cognitive decline, and higher brain reserve was a strong indicator for reversion and conversion in patients with MCI (Osone et al., 2016). Thus, the potential compensational effects due to a higher brain reserve in the aMCI corhort may limit the detection of correlations between the limbic system and EEM effect.

Using a combination of VBM and TBSS analyses, we found that both the emotional memory-related GM regions and WM connections that are anatomically inter-connected via the limbic networks were damaged in AD and aMCI patients. Besides primary degeneration of WM, such as demyelination and axonal degeneration, WM impairment can be the secondary result of primary neuronal cell body degeneration, a process known as Wallerian degeneration. We speculate that primary neuron loss of interacting and correlated emotional memory-related brain structures may cause secondary lesions to their interconnecting WM fibers, which exacerbate brain atrophy reciprocally, thus accelerating the progress of neuropathological damage of the limbic system networks and the impairment of emotional memory.

Nevertheless, there are still some limitations to the present study. Firstly, the slice thickness of raw DTI data was 4 mm, which may be too thick to view intricate structures. Secondly, as mild to moderate WMHs are common during aging, we did not consider them as exclusion criteria in the current study, and the education level of AD patients was lower than the NCs and aMCI group. However, on one hand, we have regarded these possible confounding factors as covariates, and therefore nullified them in our statistics models. On the other hand, large well-matched samples will be collected in the future to verify those findings and for further studies. Thirdly, the NC group was lack of a deep neuropsychological evaluation, and this might bring in some bias of group entrance. Fourthly, given the potential role of inflammation in psychopathology, it has been thought that chronically activated inflammatory signals in aging may contribute to increased vulnerability to neuropsychiatric and neurodegenerative disorders (Popa-Wagner et al., 2014; Sandu et al., 2015). Thus, inflammation might also be associated with the impairment of emotional memory. However, in the present study, we didn't check for systemic inflammatory markers, thus a further research is needed to focus on this potential interference. Finally, we did not distinguish the role of collection and familiarity in the present study, which may have limited the detection of EEM to some extent, and further investigation is warranted regarding these two factors.

In conclusion, our results give support that there are impairments of the limbic system networks in AD and aMCI patients, and this impairment is associated with emotional memory and the loss of EEM in AD patients. Furthermore, neuronal loss of interacting and correlated emotional memory-related brain structures may cause secondary lesions of WM fibers interconnecting these regions which exacerbate brain atrophy reciprocally, thus accelerating the progress of neuropathological damage of the limbic networks, associated with the impairment of emotional memory and loss of EEM in AD patients.

## Author contributions

YY designed the study and revised it critically for important intellectual content. XSL performed the research and drafted the manuscript. HW helped in data analyses. XHL helped draft the work. KW,YT and SZ participated in the clinical evaluation of patients. All authors read and approved the final manuscript.

### Conflict of interest statement

The authors declare that the research was conducted in the absence of any commercial or financial relationships that could be construed as a potential conflict of interest.
